# Advances in multi-omics research on viral hepatitis

**DOI:** 10.3389/fmicb.2022.987324

**Published:** 2022-09-02

**Authors:** Ze Xiang, Jiayuan Li, Di Lu, Xuyong Wei, Xiao Xu

**Affiliations:** ^1^Key Laboratory of Integrated Oncology and Intelligent Medicine of Zhejiang Province, Department of Hepatobiliary and Pancreatic Surgery, Affiliated Hangzhou First People’s Hospital, Zhejiang University School of Medicine, Hangzhou, China; ^2^Zhejiang University School of Medicine, Hangzhou, China; ^3^Westlake Laboratory of Life Sciences and Biomedicine, Hangzhou, China; ^4^NHC Key Laboratory of Combined Multi-Organ Transplantation, Hangzhou, China; ^5^Institute of Organ Transplantation, Zhejiang University, Hangzhou, China

**Keywords:** viral hepatitis, genomics, proteomics, transcriptomics, metabolomics

## Abstract

Viral hepatitis is a major global public health problem that affects hundreds of millions of people and is associated with significant morbidity and mortality. Five biologically unrelated hepatotropic viruses account for the majority of the global burden of viral hepatitis, including hepatitis A virus (HAV), hepatitis B virus (HBV), hepatitis C virus (HCV), hepatitis D virus (HDV), and hepatitis E virus (HEV). Omics is defined as the comprehensive study of the functions, relationships and roles of various types of molecules in biological cells. The multi-omics analysis has been proposed and considered key to advancing clinical precision medicine, mainly including genomics, transcriptomics and proteomics, metabolomics. Overall, the applications of multi-omics can show the origin of hepatitis viruses, explore the diagnostic and prognostics biomarkers and screen out the therapeutic targets for viral hepatitis and related diseases. To better understand the pathogenesis of viral hepatitis and related diseases, comprehensive multi-omics analysis has been widely carried out. This review mainly summarizes the applications of multi-omics in different types of viral hepatitis and related diseases, aiming to provide new insight into these diseases.

## Introduction

Viral hepatitis is a major global public health problem that affects hundreds of millions of people and is associated with significant morbidity and mortality. Five biologically unrelated hepatotropic viruses account for the majority of the global burden of viral hepatitis, including hepatitis A virus (HAV), hepatitis B virus (HBV), hepatitis C virus (HCV), hepatitis D virus (HDV), and hepatitis E virus (HEV). Despite that HAV does not develop into a chronic infection, HBV, HCV, HDV and occasionally HEV may cause chronic infections, of which HBV and HCV have a significant association with chronic incidence. Most deaths from viral hepatitis are due to HBV and HCV infections. According to the statistics, it was estimated that 296 million people were infected with hepatitis B, 58 million people were infected with hepatitis C and 1.1 million people died as a consequence of viral hepatitis infections in 2019 (Tanaka et al., 2022). In 2015, the United Nations adopted a resolution to combat viral hepatitis as part of the agenda to achieve the 2030 sustainable development goals. Subsequently, the first global strategy was developed in 2016 for the elimination of viral hepatitis (Vo Quang et al., 2021).

The advances in technology have created a variety of new fields of study, commonly referred to as omics. Omics is defined as the comprehensive study of the functions, relationships and roles of various types of molecules in biological cells. The multi-omics analysis has been proposed and considered key to advancing clinical precision medicine, including genomics, transcriptomics and proteomics, metabolomics and so on (Olivier et al., 2019). Genomics focuses on genomic DNA, the genome is usually divided into small fragments and then iteratively assembled by bioinformatics algorithms, along with gene annotation and other data analysis (Low et al., 2019). Transcriptomics can be performed to study the sum of mRNAs at a certain time point, and can also use known gene probe for specific genes (Kalisky et al., 2018). Besides, proteomics is oriented to the whole protein based on 2D-Gel and mass spectrometry. It is divided into top–down and bottom–up analysis methods. Similar to the genomics, the protein is decomposed into small peptide segments, and the known and unknown protein sequences are thus identified (Aslam et al., 2017). Through the liquid phase and mass spectrometry, metabolomics can analyze a mixture of metabolites, such as macromolecules and small molecules (Li B. et al., 2017). Technological innovation has promoted the awareness of several diseases (Wu et al., 2015, 2017a,b; Crooke et al., 2021). In general, the applications of multi-omics have provided new insights into the diagnosis, prognosis and treatment of these diseases.

To better understand the pathogenesis of viral hepatitis and related diseases, comprehensive multi-omics analysis has been widely carried out. Hence, this review summarizes the applications of multi-omics in different types of viral hepatitis and related diseases, aiming to throw light on the development of these diseases.

## Multi-omics in hepatitis A and related diseases

Hepatitis A virus is a positive-strand RNA virus, which is transmitted through the fecal oral route. HAV outbreaks are often associated with poor sanitation, overcrowding or contamination of food and water (Abutaleb and Kottilil, 2020). HAV infections in children are usually asymptomatic, but adults will present symptoms with jaundice, abdominal pain and hyperbilirubinemia.

Using genomics, Wassenaar et al. (2020) compared the intraspecific genome diversity of the single-stranded RNA(+) viruses of HAV, HCV, and HEV, and they found that these viruses all can cause hepatitis, but have no genetic similarity. Heydari et al. (2021) performed whole-genome sequencing of two patients with acute hepatitis A and plotted an HAV genome-wide phylogenetic tree. Whole-genome sequencing can clearly reveal HAV sequence. After sequence alignment, the researchers can explore its origin and spread history.

To better understand the biogenesis of quasi-enveloped HAV (eHAV) virions, McKnight et al. (2017) used proteomics quantitative analysis to successfully identify surface markers for eHAV vesicles and supported exosome-like mechanisms of eHAV outflow. In the study of duck HAV genotype 3 (DHAV-3), Liang et al. (2020) collected DHAV-3-infected duck livers for proteomic analysis, and they found that type I interferon plays an extremely important role in the pathogenic mechanism of DHAV-3. Similarly, by proteomics, DHAV-1 infection was considered to cause endoplasmic reticulum stress-induced duck embryo fibroblast cell autophagy, and proteins involved in the DHAV-1 infection process or endoplasmic reticulum stress-induced autophagy process were successfully identified (Lan et al., 2019). Infection process of HAV in the hosts associated with specific proteins will be shown using proteomics.

Kanda et al. (2015) demonstrated that epigenetic control is involved in HAV internal ribosomal entry site-dependent translation and HAV replication. It was suggested that in the clinical application of epigenetic therapy for malignant tumors, special attention should also be paid to the underlying viral disease (Kanda et al., 2015).

## Multi-omics in hepatitis B and related diseases

Hepatitis B virus is the most common cause of acute and chronic liver diseases worldwide, and approximately 4 million people are infected with HBV every year, especially in Asia and Africa (Khan A. et al., 2021). About 10% of patients infected with HBV will develop chronic infections, including liver fibrosis and cirrhosis. Each year, about one million people die from hepatitis B-related chronic liver diseases (Lu et al., 2018; Asrani et al., 2019). Most chronic hepatitis B (CHB) patients show no obvious symptoms, but as the disease progresses, they eventually develop liver cirrhosis and hepatocellular carcinoma (HCC) (Khan A. A. et al., 2021; Su et al., 2022). The applications of multi-omics can show the molecular and functional maps of HBV and related diseases.

### Genomics

By comparing the genomes of ancient African strains and HBV, Guzmán-Solís et al. (2021) found a high degree of similarity between the two viruses, which suggested that HBV may originate on the African continent and was transported to America during the transatlantic slave trade and subsequently introduced to New Spain. Next-generation sequencing methods are used to sequence concurrently, enabling us to detect any pre-existing mutations before antiviral therapy. Hence, drug resistance mutations were detected in CHB patients receiving nucleos(t)ide analog therapy using genomics (Widasari et al., 2014). Genome comparisons can enrich the discussion of HBV origin and transmission. Besides, genomics also allows us to understand HBV genotypes, quasi-species, splicing, defective HBV, virus evolution within a single host and so on.

As the DNA virus, HBV is different from other RNA hepatitis viruses since its viral genome can be integrated into the host liver cell genome. HBV integration is considered to lead to the occurrence of HCC, and the study of its structure is of great significance to the occurrence and development of HBV-related HCC. Ramirez et al. (2021) successfully fabricated a panel of HBV-targeting biotinylated oligonucleotide probes, and they described the structure and transcriptional signatures of integrated HBV in different HCC cell lines. At the junctions between chromosomes, five chromosomal translocations integrating HBV DNA were found, and many integrations and translocations were transcriptionally silent, which further revealed the possible mediating mechanism of HBV-related HCC (Ramirez et al., 2021). To clearly describe the structure of HBV integration, Péneau et al. (2022) found that clonal selection for HBV integration may be associated with two mechanisms that lead to HCC through long-read sequencing or Bionano whole genome mapping. The first possible mechanism is that the integration of viral enhancers near the cancer driver gene may lead to overexpression of the oncogene, and the second possible mechanism is that frequent chromosomal rearrangements at the HBV integration site can cause changes in the distance of the cancer driver gene. Therefore, HBV integration is thought to have the ability to predict HBV-associated patients with HCC and has a certain clinical value (Péneau et al., 2022). In addition, the structure of HBV isolated from HCC patients was also determined, and it was found that HBV immune escape mutants may be an important factor in the occurrence and development of HCC (Lin et al., 2002). The applications of genomics in HBV-related diseases mainly focus on HBV-induced HCC. Gene expression profiling can facilitate the discovery of diagnostic and prognostic markers for HBV-related HCC.

Advances in genomics have deepened the understanding of the diagnosis, prognosis of HBV-related HCC. The detailed genetic analysis of liver tissue provides important information for tumorigenesis and progression (Dhanasekaran et al., 2019; Wu Y. et al., 2020; Wei et al., 2022). The findings of genomics research may promote the progress of individualized management of HCC, thereby innovating therapeutic methods.

### Proteomics

The applications of proteomics are of great significance in hepatitis B and related diseases (Table 1). Proteomics can help reveal the origin and development of HBV. Krause-Kyora et al. (2018) showed that HBV has been circulating in European populations for over 7,000 years through proteomics.

**TABLE 1:** The applications of proteomics in hepatitis B and related diseases.

Authors	Diseases	Biomarkers	Role
Krause-Kyora et al., 2018	HBV infection	–	Proteomics showed the origin and development of HBV.
Zeyen et al., 2020	HBV infection	Coat protein complex II, Sec24A and Sec23B	Hepatitis B subviral enveloped particles utilize the coat protein complex II for intracellular transport by selectively utilizing Sec24A and Sec23B.
Yan et al., 2018	HBV infection	RSK2	RSK2 plays a role in HBx enhancing HBV replication.
Murphy et al., 2016	HBV infection	SMC5/6	SMC5/6 can suppress HBV replication by inhibiting HBV gene expression.
Xie et al., 2011	HBV infection	Cyclosporine A	Cyclosporine A has an inhibitory effect on HBV replication.
Zhou et al., 2021	HBV infection	Pin1	Pin1 is an interactor that binds to the transactivation domain of HBx.
Zhao et al., 2020	HBV infection	S100 proteins	High expression of S100 proteins is related to the transmission of HBV in the placenta.
Chen et al., 2021	HBV infection	VWF and C8B	VWF and C8B have the potential to distinguish HBV infection genotype B and genotype C.
Tsai and Hsiao, 2017	HBV infection	YLWEWASVR	The peptide YLWEWASVR derived from hepatitis B surface antigen was confirmed as a biomarker for the diagnosis of HBV infection.
Liu et al., 2019	CHB infection	Fibronectin	Fibronectin levels in plasma have been demonstrated to be a predictor of HBsAg clearance
McBrearty et al., 2021	CHB infection	Short-chain fatty acids	Short-chain fatty acids are able to prevent CHB from progressing to HCC.
He et al., 2016	HBV-related cirrhosis	ACY1	ACY1 autoantibodies were considered as biomarkers to differentiate HBV-related cirrhosis and CHB patients.
Dai et al., 2019	CHB with different fibrosis stages	Ficolin-2 and carboxypeptidase B2	The expression of ficolin-2 (FCN2) and carboxypeptidase B2 (CPB2) was different in CHB patients with different fibrosis stages.
Katrinli et al., 2016	Different stages of fibrosis	HIF-1α	The interaction between HBx and HIF-1α may be a novel target pathway for therapies.
Kan et al., 2017	HBV-related fibrosis	CAT, BLVRB, NXN, PRDX1 and IDH1	CAT, BLVRB, NXN, PRDX1, and IDH1 were also identified as possible drug and therapeutic targets for the detection of HBV-related fibrosis.
Ye et al., 2020	HBV-associated fibrosis	AAV shRNAs	AAV shRNAs can effectively regulate HBV-associated fibrosis by reducing oxidative stress, inflammation, and activating the PPAR signaling pathway.
Wu D. et al., 2020	HBV-ACLF	Plasminogen	Plasminogen can be used as a prognostic marker for HBV-ACLF.
Sun et al., 2019	HBV-ACLF	–	The hematological dysfunction of HBV-ACLF patients was revealed and a diagnostic and prognostic model established.
Zhou et al., 2017	HBV-ACLF	–	Six novel HBV-ACLF candidate biomarker may provide basic information for the study of HBV-ACLF biomarkers.
Wei et al., 2016	HBV-induced HCC	ARFIP2 and ANXA1	ARFIP2 and ANXA1 are potential biomarkers to differentiate HBV genotype B and C-induced HCC.
Xu et al., 2017	HBx-mediated HCC	–	HBx/CDC42/IQGAP1 signaling pathway may play an important role in HBx-mediated HCC.
Zhang et al., 2007	HBV-infected G1 tumors	Proteasome activator subunit 1 and DJ-1	Proteasome activator subunit 1 and DJ-1 were found to be downregulated in HBV-infected G1 tumors.
Lin et al., 2022	HBV-related HCC	–	An LGPI model was constructed by screening differential proteins in non-tumor liver tissue and HCC liver tissue.
Chai et al., 2021	HBV-related HCC	H2BK120ac, H3.3K18ac and H4K77ac	H2BK120ac, H3.3K18ac and H4K77ac were confirmed to be significantly associated with HBV-related HCC prognosis.
Gao et al., 2019	HBV-related HCC	PYCR2 and ADH1A	PYCR2 and ADH1A were associated with HBV-related HCC prognosis.
Li et al., 2019	HBV-related HCC	CREB1	HBx-CTTN interaction can promote HCC proliferation and migration through CREB1.

The applications of proteomics provide new strategies for the occurrence, progression and replication of HBV. Based on yeast proteomics, Zeyen et al. (2020) found that hepatitis B subviral enveloped particles utilize the coat protein complex II component for intracellular transport by selectively utilizing Sec24A and Sec23B. Based on isobaric tags for relative and absolute quantitation (iTRAQ) quantitative comparative proteomics, RSK2 was identified as a novel host protein that plays a role in HBx enhancing HBV replication (Yan et al., 2018). Using the substrate capture proteomics, Murphy et al. (2016) showed that the main function of HBx is to degrade SMC5/6, which can suppress HBV replication by inhibiting HBV gene expression. Xie et al. (2011) found that HBx has a promoting effect on HBV replication, while they confirmed that cyclosporine A has an inhibitory effect on HBV replication. Pin1 is considered to be an interactor that binds to the transactivation domain of HBx, suggesting the potential relationship between Pin1 and the function of HBx in HBV replication (Zhou et al., 2021). In addition, through iTRAQ proteomic analysis, Zhao et al. (2020) found that the high expression of S100 proteins is related to the transmission of HBV in the placenta, which provides new insight into the mother-to-infant transmission of HBV.

In addition to the traditional HBV markers HBsAg and anti-HBs, the applications of proteomics have also expanded the development of diagnostic markers. Two differential proteins, VWF and C8B were considered to have the potential to distinguish HBV infection genotypes B and C and could provide precise guidance for HBV genotyping (Chen et al., 2021). The peptide YLWEWASVR derived from the hepatitis B surface antigen was confirmed as a biomarker for the diagnosis of hepatitis B virus infection (Tsai and Hsiao, 2017). Moreover, the use of proteomics/genomics databased in the identification of the HBV receptor in 2012, which is considered as one of the most important discoveries related to HBV in the last decade (Yan et al., 2012).

In CHB patients, fibronectin levels in plasma have been demonstrated to be a predictor of HBsAg clearance (Liu et al., 2019). Long-term HBV infection has been shown to lead to cellular proteome remodeling, which can mediate the pathological effect (Zai et al., 2022). Through the mass spectrometry-based proteomic analysis, McBrearty et al. (2021) found that short-chain fatty acids can prevent CHB from progressing to HCC.

In recent years, the applications of proteomics in HBV-related cirrhosis have also been reported. Autoantibodies recognized aminoacylase-1 (ACY1) were considered biomarkers to differentiate HBV-related cirrhosis and CHB patients by serum proteomic detection (He et al., 2016). Proteomics also provides new ideas for the diagnosis, prognosis and treatment of HBV-induced fibrosis. Through serum proteomics analysis, Dai et al. found that the expression of ficolin-2 (FCN2) and carboxypeptidase B2 (CPB2) was different in CHB patients with different fibrosis stages, indicating the diagnostic value of FCN2 and CPB2 (Dai et al., 2019). At different stages of fibrosis, Katrinli et al. (2016) observed changes in the glycolytic pathway caused by the presence of HBx, so the interaction between it and HIF-1α may be a novel target pathway for therapies. Kan et al. (2017) screened 28 HBV-specific proteins by comprehensive proteomics and transcriptomics, and they emphasized the critical role of oxidative stress in HBV-related liver fibrosis. Catalase (CAT), Biliverdin Reductase B (BLVRB), Nucleoredoxin (NXN), Peroxiredoxin 1 (PRDX1), and Isocitrate Dehydrogenase [NADP(+)] 1 (IDH1) were also identified as possible drug and therapeutic targets for the detection of HBV-related fibrosis. AAV shRNAs were found to effectively regulate HBV-associated fibrosis by reducing oxidative stress, inflammation, and activating the PPAR signaling pathway (Ye et al., 2020).

Proteomics also plays an important role in HBV-related liver failure. Wu D. et al. (2020) used TMT-labeled quantitative proteomics to find that plasminogen can be used as a prognostic marker for HBV-ACLF. Sun et al. (2019) revealed the hematological dysfunction of HBV-ACLF patients through targeted proteomics and established a diagnostic and prognostic model. Quantitative proteomics analyses have identified six novel HBV-ACLF candidate biomarkers, which may provide basic information for the study of HBV-ACLF biomarkers (Zhou et al., 2017).

As HBV is a risk factor for the development of HCC, the applications of HBV-related HCC proteomics have also been paid more and more attention. Wei et al. (2016) concluded that ARFIP2 and ANXA1 are potential biomarkers to differentiate HBV genotype B and C-induced HCC through quantitative proteomic analysis. With the altered protein expression during the progression of HBV-related HCC, some proteins can be considered potential biomarkers for diagnosis and therapy (Jiang et al., 2019). Through the quantitative proteomics, Xu et al. (2017) found that the HBx/CDC42/IQGAP1 signaling pathway may play an important role in HBx-mediated HCC. Similarly, proteasome activator subunit 1 and DJ-1 were found to be downregulated in HBV-infected G1 tumors, revealing their possible mediating mechanisms (Zhang et al., 2007). Lin et al. (2022) constructed an LGPI model by screening differential proteins in non-tumor liver tissue and HCC liver tissue, predicting the overall survival and prognosis of patients with HBV-related HCC. By quantitative proteomics, H2BK120ac, H3.3K18ac and H4K77ac were confirmed to be significantly associated with HBV-related HCC prognosis (Chai et al., 2021). Gao et al. (2019) screened out two HCC metabolic reprogram prognostic markers associated with HBV-related HCC, PYCR2, and ADH1A. HBx-CTTN interaction can promote HCC proliferation and migration through CREB1, and the HBx/CTTN/CREB1 axis was considered a potential new therapeutic target for HCC (Li et al., 2019).

### Metabolomics

Through the differential metabolomic analysis, Luteolin-7-*O*-glucoside was confirmed to inhibit HBsAg and HBV replication through mechanisms involving mitochondria (Cui et al., 2017). Yu et al. (2022) focused on metabolic changes during HBV replication and infection, and they found that the high levels of amino acids depletion and phosphatidylcholines and lysophosphatidylcholines biosynthesis play important roles in the pathogenesis of HBV infection. Through the metabolomic analysis, Hu et al. (2021) found that HBP-mediated *O*-GlcNAcylation can positively regulate the host’s antiviral response to HBV.

Combined with serum-targeted metabolomics, the metabolic signature of CHB infection progression was further revealed (Schoeman et al., 2016). By analyzing metabolomics data at different stages in patients with CHB, Nguyen et al. (2021) found that ammonia detoxification, glutamine and glutamate metabolism, methionine metabolism, branched-chain amino acid imbalance, and disorders of the tricarboxylic acid cycle are influencing factors in the progression of patients with CHB. Combined with the gut microbiome and metabolome, Sun et al. (2021) provided new insights into bile acid metabolic pathways in patients with CHB.

Through serum metabolomics, Nie et al. (2014) found that 17 metabolites were associated with the prognosis of HBV-related acute-on-chronic liver failure (HBV-ACLF), providing information for markers for the diagnosis and prognosis of HBV-ACLF. Lian et al. (2016) analyzed serum samples from HBV-ACLF, HBV-related chronic liver failure (HBV-CLF) and healthy populations, and they found that phosphatidylcholines, lysophosphatidylcholines and conjugated bile acids (GCDCA, GUDCA) metabolites may act as markers for ACLF and CLF diagnosis and provide new insights into the pathogenesis of ACLF and CLF.

Through metabolomics, amino acid imbalance metabolism was thought to play an important role in the development and progression of HBV-related HCC (Huang et al., 2020). Compared with HBV-infected patients, HBV-related HCC patients have lower levels of metabolite lysophosphatidylcholines in their blood, which may serve as a clinical diagnostic marker for HCC (Li et al., 2021). Through serum metabolomics, Cai et al. (2020) discovered enzymes associated with HBV-related HCC diagnosis and prognosis. Through the genetic screening, combined with RNA-seq and metabolomic analysis, Chen et al. (2022) found the joint effect of PSTK as a resistance medium for targeted therapy of HCC cells, suggesting an ideal treatment method for HBV-related HCC.

### Transcriptomics

Transcriptomics functions in the study of HBV and related diseases. Using RNA-seq transcriptomics, König et al. (2019) demonstrated that HBV does not induce significant gene expression changes in HepG2-NTCPsec+ and that HepG2-NTCPsec + cells support a net amplification of the HBV genome, leading to the development of a new model of HBV infection. Hou et al. (2017) conducted an in-depth transfer group analysis of formalin-fixed paraffin-embedding liver biopsy in the clinical stage. They found that viral load and liver injury are associated with the fluctuations that coincided with those of the liver transcriptome (Hou et al., 2017). Using transcriptomic and proteomic methods, the RIG-I-like receptor signaling pathway was confirmed to be the main signaling pathway for changes in HBV-related fibrosis (Kan et al., 2017). In addition, Li et al. (2022) found that HBV exacerbation-induced immune dysregulation disorder is the underlying mechanism identified in HBV-ACLF through mRNA sequencing of peripheral blood mononuclear cells in patients.

## Multi-omics in hepatitis C and related diseases

Hepatitis C virus virions are spherical and are single-stranded positive-stranded RNA viruses. HCV can often cause hepatitis C infection. According to estimates, approximately 71 million people worldwide suffer from chronic hepatitis C virus (CHC) infection (Rabaan et al., 2020). CHC infection is associated with advanced liver disease and can induce hepatocellular carcinoma, which will cause many extrahepatic manifestations. The applications of multi-omics also help researchers understand HCV and related diseases deeply.

### Genomics

Through functional genomics, Li et al. (2016) found that *E*-cadherin is a mediator of HCV entry into host cells and is closely related to HCV-induced epithelial-mesenchymal transition. Takagi et al. (2021) revealed the sequence of HCV-G4-KM long clones by sequencing the HCV-G4-KM long clones in mouse serum, and they proved that the sequence of HCV-G4-KM long NAs plays an important role in infectious cloning. By combining proteomics and genomics, Ramage et al. (2015) demonstrated the role of HCV between the infectious process and the host, and they explored the mechanism by which HCV affects the function of the infected host. In addition, functional genomics has been used to explore the interaction between HCV and miRNA and also demonstrated the HCV-mediated pathogenesis (Li Q. et al., 2017). Genomics can reveal the process of HCV entering and infecting host cells.

### Proteomics

Proteomics matters in hepatitis C and related diseases (Table 2). Proteomics can show the process of HCV assembly, host cell entry and replication. Kumar et al. (2019) reported that MARCH8 catalyzes polyubiquitination of K63-linked HCV non-structural 2 proteins, followed by ESCRT recruitment and HCV envelope. Proteomics of HCV virions determined an essential role for the nucleoporin Nup98 in virus morphogenesis (Lussignol et al., 2016). HCV was confirmed to enter hepatocytes through the CD81 receptor complex calpain-5 and CBLB (Bruening et al., 2018). Gerold et al. (2015) identified serum response factor binding protein 1 by quantitative proteomics, which can be recruited to CD81 during HCV uptake and support HCV infection in HCC cells and primary human hepatocytes. Borawski et al. (2009) demonstrated that both class III phosphatidylinositol 4-kinases α and β are novel host factor regulators of HCV replication. In addition, HCV was reported to induce lipid rafts to localize to autophagosomes, thereby mediating HCV RNA replication (Kim et al., 2017).

**TABLE 2:** The applications of proteomics in hepatitis C and related diseases.

Authors	Diseases	Biomarkers	Role
Kumar et al., 2019	HCV infection	MARCH8	MARCH8 catalyzes polyubiquitination of K63-linked HCV non-structural 2 proteins, followed by ESCRT recruitment and HCV envelope.
Lussignol et al., 2016	HCV infection	Nup98	Proteomics of HCV virions determined an essential role for the nucleoporin Nup98 in virus morphogenesis.
Bruening et al., 2018	HCV infection	CD81 receptor complex calpain-5 and CBLB	HCV was confirmed to enter hepatocytes through the CD81 receptor complex calpain-5 and CBLB.
Gerold et al., 2015	HCV infection	Serum response factor binding protein 1	Serum response factor binding protein 1 can be recruited to CD81 during HCV uptake.
Borawski et al., 2009	HCV infection	Class III phosphatidylinositol 4-kinases α and β	Both class III phosphatidylinositol 4-kinases α and β are novel host factor regulators of HCV replication.
Kim et al., 2017	HCV infection	–	HCV was reported to induce lipid rafts to localize to autophagosomes, thereby mediating HCV RNA replication.
Gangadharan et al., 2012	HCV-associated liver fibrosis	–	20 novel biomarkers of HCV-associated liver fibrosis were identified to assess the degree of liver fibrosis.
Cheung et al., 2010	HCV-related liver fibrosis and cirrhosis	G3BP	G3BP can be used as a marker for HCV-related liver fibrosis and cirrhosis.
Yang et al., 2011	HCV-related liver fibrosis	C4-A and inter-α-trypsin inhibitor heavy chain H4	C4-A and inter-α-trypsin inhibitor heavy chain H4 were screened to predict HCV-related liver fibrosis.
Lee et al., 2006	HCV-related HCC	C3a	Complement C3a as a candidate biomarker for HCV-related HCC.
Malov et al., 2021	HCV-related HCC	Glypican-3 and osteopontin	Glypican-3 and osteopontin, could serve as HCV-associated HCC markers.

Gangadharan et al. (2012) identified 20 novel biomarkers of HCV-associated liver fibrosis to assess the degree of liver fibrosis. Cheung et al. (2010) found that G3BP can be used as a marker for HCV-related liver fibrosis and cirrhosis. Through the serum proteomics, C4-A and inter-α-trypsin inhibitor heavy chain H4 were screened to predict HCV-related liver fibrosis (Yang et al., 2011). The development of biomarkers for HCV-associated fibrosis and cirrhosis were also explored using genomics.

Diagnostic and prognostic biomarkers of HCV-related HCC can be discovered by proteomics. Lee et al. (2006) identified complement C3a as a candidate biomarker for HCV-related HCC by proteomics. Malov et al. (2021) confirmed that heparin binds to growth factors, glypican-3 and osteopontin could serve as HCV-associated HCC markers. The screening of specific proteins is conducive to accurate diagnosis and prognosis in patients with HCV infection and related diseases.

### Metabolomics

Through the comprehensive metabolomics analysis, Fitian et al. (2014) identified overall metabolic disorders in patients with HCV-associated HCC and cirrhosis, and they hypothesized that abnormal dicarboxylic acid metabolism, enhanced bile acid metabolism, and elevated fibrinogen-cleaved peptides might be the signs of liver cirrhosis. Shanmuganathan et al. (2021) compared the ability of multisegment injection-capillary electrophoresis-mass spectrometry and nuclear magnetic resonance to characterize the serum metabolome, and they found that both instrumental techniques can quickly and reliably quantify serum metabolites in large-scale metabolomics research with the good overlap of biomarker replication. Therefore, metabolomics can show the metabolic process of HCV infection and its associated diseases.

### Transcriptomics

Through the genome-wide miRNA functional screening and transcriptomic analysis, Sodroski et al. (2019) generated a comprehensive map of HCV-miRNA interactions. They found that inhibition of key host restriction factors mediates the proviral effects of miR-135a on HCV transmission (Sodroski et al., 2019). Through the comprehensive functional genomics analysis, miR-25, let-7, and miR-130 families were also proved to inhibit the necessary HCV cofactors, thus limiting HCV infection in multiple stages (Li Q. et al., 2017). Hence, cellular microRNAs have been shown to regulate HCV infection by acting directly on the viral genome or indirectly on virus-associated host factors.

## Multi-omics in hepatitis D and related diseases

Hepatitis D virus is only found in humans currently. It is a satellite virus, which is assembled, released and entered by the envelope protein of HBV. It is the smallest known RNA virus, encoding a single protein.

Using meta-transcriptomics, Wille et al. (2018) identified the genome of a novel HDV in duck. Sequence analysis showed that HDVs share a common secondary structure. The predicted viral protein shares a 32% amino acid similarity with the small delta antigen of HDV, which contains a distinct phylogenetic lineage. The discovery of avian influenza virus-like pathogens helps us better understand the origin of HDV and subviral pathogens (Wille et al., 2018). Taking meta-transcriptomic data, Chang et al. (2019) found that highly differentiated HDV-like viruses also exist in fish, amphibians and invertebrates. None of these novel HDV-like infections is associated with other hepatitis virus infections, supporting the idea that the HDV-HBV association may be unique to humans (Chang et al., 2019). To summary, transcriptomics can reveal the diversity and host range of HDV, and also indicate the origin and evolutionary history of HDV.

## Multi-omics in hepatitis E and related diseases

Hepatitis E virus (HEV) is an important zoonotic virus that can infect various hosts. It has 7 main genotypes. Patients with HEV infection are mostly asymptomatic, some patients will present jaundice and symptoms of acute hepatitis (Desai, 2020). Besides, HEV infection can also cause many extrahepatic manifestations (European Association for the Study of the Liver, 2018; Wu et al., 2021).

The study by Shen et al. (2014) employed a comparative gel proteomics approach to investigate the changes in A549 cell proteins following *in vitro* HEV exposure, which was beneficial for the study of the interaction between HEV and host cells. Three different strains of porcine HEV were identified by Rogée et al. (2015). They revealed the process by which HEV damages cells, providing important evidence for the replication factors and related pathogenesis of HEV (Rogée et al., 2015). Through serum metabolomics, it was demonstrated that dynamic changes in serum metabolites were associated with AHE infection and severity (Wu et al., 2022b). Through the meta-transcriptomic, Zhang et al. (2019) determined the HEV virus subtypes in broilers and further proved by the phylogenetic analysis that the avian HEV identified in the study is a novel subtype of genotype 3 avian HEV. Thus, transcriptomics provides complete genomic data on the evolutionary relationships of avian HEV, which helps us further understand the evolution of HEV. Besides, Wu et al. (2022a) performed the 16S ribosomal ribonucleic acid gene sequencing, and they found that gut microbiota dysbiosis is associated with plasma levels of Interferon-γ and viral load in patients with acute hepatitis E infection. Overall, the investigation on HEV infection and related diseases using multi-omics are less, which requires more efforts.

## Conclusion and perspectives

**FIGURE 1:**
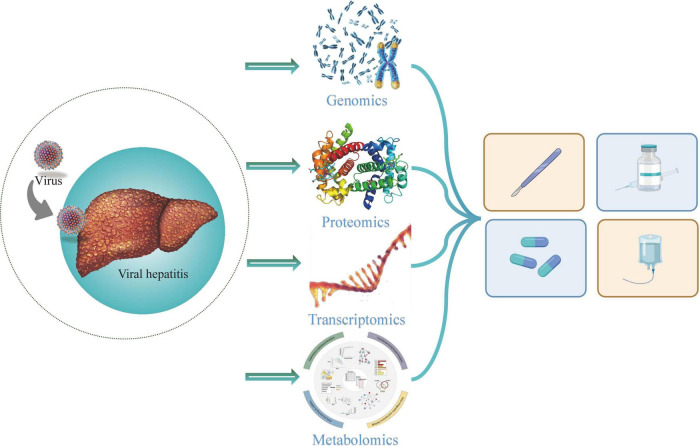
Multi-omics in viral hepatitis.

In conclusion, the applications of multi-omics have shown the origin and development of the hepatitis virus and provided new strategies for the diagnosis, prognosis and treatment of viral hepatitis and related diseases (Figure 1). There are many multi-omics studies on HBV infection, HCV infection and related diseases, several biomarkers were found and more correlations were revealed. Nevertheless, the accuracy of these screened biomarkers for the diagnosis and prognosis in patients still needs to be discussed. Moreover, multi-omics studies on other hepatitis are inadequate, and more efforts should be made.

Besides, multi-omics applications are not limited to genomics, proteomics, metabolomics and transcriptomics, and other omics are also developing, including radiomics, viromics, and so on. The joint application of these omics is believed to provide new insight into viral hepatitis and related diseases.

## Author contributions

ZX and JL had the idea for the manuscript. ZX and DL performed the literature search and data analysis. XX and XW drafted and critically revised the work. All authors contributed to the article and approved the submitted version.
